# The prevalence of chlamydia trachomatis among patients with acute conjunctivitis in Kasr Alainy ophthalmology clinic

**DOI:** 10.11604/pamj.2014.17.151.3818

**Published:** 2014-03-03

**Authors:** Maha Abdelrahman Mowafy, Nagwa Eid Saad, Hala Mohamed El-Mofty, Mervat Gaber ElAnany, Marwa Sayed Mohamed

**Affiliations:** 1 Family Medicine Department, Faculty of Medicine, Cairo University, Cairo, Egypt; 2 Ophthalmology Department, Faculty of Medicine, Cairo University, Cairo, Egypt; 3 Clinical and chemical pathology Department, Faculty of Medicine, Cairo University, Cairo, Egypt

**Keywords:** Chlamydia Trachomatis, conjunctivitis, trachoma, blindness

## Abstract

**Introduction:**

Trachoma is a leading cause of avoidable blindness and endemic conjunctivitis in 57 countries. It infects approximately 84 million people globally, and continues to threaten over 10% of the world's population with the risk of blindness.

**Methods:**

This is a cross sectional descriptive study assessing patients presenting with acute conjunctivitis. A full history was taken from patients followed by examination of both eyes. A conjunctival swab was taken and a sample of tears was collected and handled at the central laboratory unit at Kasr AlAiny hospital for culture and sensitivity of the swab and ELISA for tears searching for Immunoglobulin G and Immunoglobulin M of chlamydia trachomatis.

**Results:**

The prevalence of bacterial conjunctivitis encounted for 45.7% and non-bacterial 54.3% of the studied group. The anti-chlamydial antibodies were positive in the tears of 31.1% of patients. While the other bacterial organisms responsible for 14.6%.

**Conclusion:**

The study concluded that trachoma accounts for one third of the cases of acute conjunctivitis while the other bacterial organisms responsible for about 14.6%. More than half of the cases have other causes as viral, allergic, mechanical or chemical induced conjunctivitis.

## Introduction

Chlamydia eye disease is one of the earliest known eye infections, having been identified in Egypt as early as 15 B C. Trachoma became a problem as people moved into crowded towns where hygiene was poor. It became a particular problem in Europe in the 19th Century after the Egyptian Campaign (1798-1802) and the Napoleonic Wars (1798-1815). Most victims of trachoma live in underdeveloped and poverty-stricken countries in Africa, the Middle East and Asia [1].

The World Health Organization (WHO) estimates that globally about 314 million people are visually impaired and it has been estimated that over 80% of global visual impairment is preventable or treatable. The major causes of blindness include cataract, uncorrected refractive errors, glaucoma, age-related macular degeneration, corneal opacities, diabetic retinopathy, eye diseases in children and trachoma [2].

Many risk factors are recognized to be associated with active trachoma. Low-economic status, crowding, lack of hygiene, and behavioral attitudes are known to be key epidemiological determinants of active trachoma. The presence of flies remains very significant for trachoma occurrence. A dirty face was significantly linked with both active and intense inflammatory trachoma children who do not wash their faces regularly may be at greater risk of trachoma [3].

Evidence of widespread distribution of trachoma in Egypt had not been clarified as previous surveys were limited to individual communities which may not have been representative of the general population. Thus this is a cross sectional descriptive study aimed to detect the magnitude of chlamydia trachomatis eye infection in Kasr Al Ainy ophthalmology outpatient clinics as a preventable cause of blindness. Taking in consideration that Kasr Alainy outpatient clinics receive patients from different parts of the country including referred cases from urban and rural communities and represents all age groups [4].

## Methods

This is a cross sectional descriptive study conducted at Kasr Alainy ophthalmology outpatient clinics, Faculty of Medicine, Cairo University (referral hospital). It started on April 2012 and completed by October 2012. The ophthalmology outpatient clinics consists of two clinics A and B with total number of cases around 270 cases per day referred from different governorates. A Convenient sample of 302 patients was collected over a period of 6 months duration from both clinics. The study targeted patients of both sexes presenting with symptoms of acute conjunctivitis (eye discharge, lacrimation, pain, itching or redness) regardless of their age, and with acute eye symptoms not related to trauma. Exclusion criteria included: Lacrimal passage obstruction, postoperative patients, and other causes of red eye apart from acute conjunctivitis (eg. Iritis,corneal ulcer, etc.)

### Microbiology workup

A conjunctival swab sample was collected for culture and sensitivity then transferred to the Microbiology unit of the central laboratory of Cairo University Hospitals for culture and sensitivity. Tears sample collected by capillary tube[5] after applying a drop of artificial tears (previously tested by ELISA for trachoma antibodies and results were zero for both Immunoglobulin G and Immunoglobulin M) then collected in 0.5 ml sterile tubes for detecting trachoma antibodies (Immunoglobulin M and Immunoglobulin G) by ELISA in tears. It was found that in normal individuals the mean Immunoglobulin A level was 30.7 mg/100 ml. Immunoglobulin G level was less than 1 mg/100 ml. Immunoglobulin M value was less than 1 mg/100 ml. IgD could not be detected [6]. Thus the cutoff number for positive Immunoglobulin G and Immunoglobulin M was equal or more than 10mg/L.

**Ethical consideration:** approval of Ethical Committee of both the Family Medicine department & Faculty of Medicine were obtained and participants gave informed written consent.

## Results

**Figure 1:**
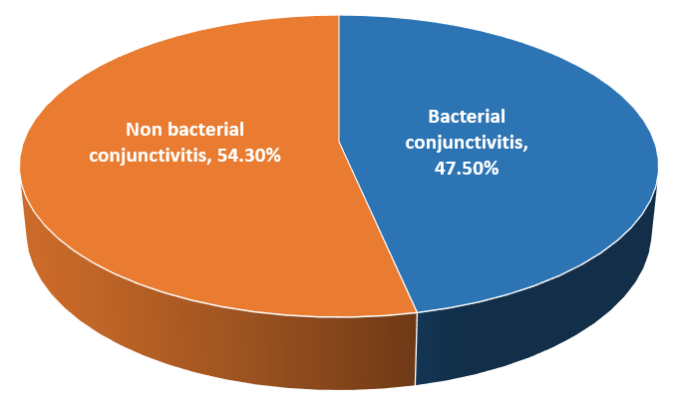
Prevalence of bacterial conjunctivitis among the studied group

The study results showed that bacterial conjunctivitis encounter 45.7% and non-bacterial encounter 54.3% of our studied group (Figure 1). The bacterial infection is divided 31.1% chlamydia trachomatis and 14.6% other bacterial organisms. The mean age of the studied group was 44.4 ±14.2 standard deviation. The maximum age was 80 years while the minimum was 14 years. Around three-quarters of the study sample were females representing 77.2% of the study sample, while males were 22.8%. Illiterates encounter 65.2% of the study sample, while a total of 76.8% of the sample were not working. Regarding the residency around half of the sample 46.7% lives in urban and half 53.3% live in rural areas. The average income was less than 500 pounds in 64.9% and more than 500 pounds with 35.1%.

As showed in Table 1; the main presenting symptom among patients was lacrimation 26.8% followed by discharge 25.8% respectively. Pain and redness were almost equally distributed 21.5% and 21.9% respectively. The least presenting complaint was itching 1% among the studied sample. Half of the studied group had associated symptoms with the main complaint 50.3% mainly in the form of redness, lacrimation and foreign body sensation respectively. It is to be noted from Table 1 that; 78.1% of patients presented with bilateral eye affection in comparison to 21.9% who had unilateral affection. Also, it was found that about half of the studied sample 49.3% had recurrent symptoms; while the other half 50.7% were presenting for the first time. Among the 149 patient with recurrence of symptoms 32.1% recurred during a period of less than six months while 17.2% of patients stated recurrence over a period of time more than 6 months. Of the studied sample 50.3% stated previous use of topical eye preparations, 15.2% had a history of previous eye operations and only 0.7% gave history of previously done laboratory investigations. Among the studied group 33.4% suffer from chronic disease mainly in the form of hypertension and diabetes respectively. It is noteworthy that only 2% of patients mentioned history of allergy and only 0.7% of patients have a family member complaining of the same symptoms.

**Table 1:** Main complaint and medical history of the study sample

Medical history	No	%
**Main complaints**		
Lacrimation	81	26.8
Discharge	78	25.8
Redness	66	21.9
Pain	65	21.5
Foreign body sensation	9	3
Itching	3	1
**Associated ophthalmological symptoms**	152	50.3%
**affected side**		
Unilateral	66	21.9
Bilateral	236	78.1
Recurrent symptoms	149	49.3
previous topical treatment	152	50.3
Previous eye operations	46	15.2
Previous laboratory eye investigations	2	0.7
Chronic disease	101	33.4
Allergy	6	2
Positive family symptoms	2	0.7

The results of the culture of conjunctival swab of the sample in Table 2 showed that there was no growth in the majority of cases 85.4%, and positive culture was only found in 14.6%. The anti-chlamydia trachomatis Immunoglobulin G in tears was found to be positive in 12.6% and negative in 87.4% of patients. On the other hand, anti- chlamydia trachomatis Immunoglobulin M in tears was positive in 23.2% and negative in 76.8% patients. The total number of patients who had positive anti-chlamydia antibodies is 31.1% either having positive Immunoglobulin G or Immunoglobulin M.

**Table 2:** Laboratory investigations of the study sample

Investigations	No	%	Total
Culture			
No growth*	258	85.4	302
CONS *(Coagulase*-*negative staphylococci)*	27	8.9	302
Staphylococcus aureus	10	3.3	302
Streptococcus pneumonia	3	1	302
Moraxella catarrhalis	1	0.3	302
MRSA(methicillin resistant staphylococcus aureus)	2	0.8	302
Pseudomonas aeruginosa	1	0.3	302
**Anti-chlamydial Immunoglobulin G in tears**			
Positive	38	12.6	
**Anti-chlamydial Immunoglobulin M in tears**			
Positive	70	23.2	
**Positive ELISA for chlamydia**	94	31.1	
**Mixed infection (positive culture and chlamydia)**	14	4.6	

* No growth could be due to chlamydia trachomatis, viral or allergic

Table 3 shows that there is a significant relationship (P value= 0.02) between Immunoglobulin G state and sex; as anti-chlamydial Immunoglobulin G in tears was positive in 38 cases of which 63.2% among females and 36.8% among males. Also, anti-chlamydial Immunoglobulin M in tears was positive in 70 cases of which 80% were females and 20% were males. The relation between Immunoglobulin G and residency and can be concluded that 63.2% of cases live in rural area have positive anti-chlamydial Immunoglobulin G antibody while 36.8% of the positive Immunoglobulin G live in urban areas. Positive anti-chlamydial Immunoglobulin M in tears was found in 70 cases of which 48.6% live in rural areas while 51.4% live in urban areas.

**Table 3:** Correlation between risk factors and anti-chlamydial Immunoglobulin G and Immunoglobulin M in tears

Risk factors	Positive Immunoglobulin G%	P- value*	Positive Immunoglobulin M%	P- value*
**Sex**				
Male	36.8	0.02*	20	0.51
Female	63.2	0.02*	80	0.51
**Residency**				
Rural	63.2	0.19	48.6	0.36
Urban	36.8	0.19	51.4	0.36
**Income**				
Less than 500	68.4	0.62	74.3	0.06
More than 500	31.6	0.62	25.7	0.06
**Education**				
Illiterate	68.4	0.65	75.7	0.03*
Non-illiterate	31.6	0.65	24.3	0.03*
**Main complaint**				
Pain	10.5	0.08	25.7	0.48
Lacrimation	23.7	0.08	24.3	0.48
Discharge	42.1	0.08	21.4	0.48
Redness	21.1	0.08	27.1	0.48
Foreign body sensation	0.0	0.08	1.5	0.48
Itching	2.6	0.08	0.0	0.48

* P value considered significant if ≤0.05

It is to be noted that positive Immunoglobulin G and Immunoglobulin M were found more in the study sample where the income was less than 500 68.4% and 74.3% respectively. The table also shows that 68.4% of the positive Immunoglobulin G was illiterate. There was a statistical significance (P value= 0.03) between anti-chlamydial Immunoglobulin M in tears and the educational state in 75.7% of the positive Immunoglobulin M cases.

Among the 38 cases that show positive anti-chlamydial Immunoglobulin G in tears the major presentation was discharge by 42.1% followed by lacrimation in 23.7%, conjunctival congestion (red eye) in 21.1%, pain in 10.5% and itching in 2.6%. Among the 70 cases that show positive anti-chlamydial Immunoglobulin M in tears the major presentation was conjunctival congestion (red eye) in 27.1% followed by pain in 25.7%, lacrimation in 24.3%, discharge in 21.4% and foreign body sensation in 1.5% of cases. Among the positive Immunoglobulin G cases Symptoms of acute conjunctivitis were recurrent in 42.1% of the sample and symptoms of acute conjunctivitis for the first time in 57.9% of cases.

## Discussion

In this study the prevalence of trachoma was 31.1% mainly among rural residents. The Nile Delta of Egypt represents a unique environment for trachoma to persist. Although economic improvements in last decade have affected even the poorest rural environments, the poor hygienic conditions still the primary factor in trachoma transmission [4]. In this study more than 50% of the studied group lives in rural area and this goes hand in hand with Ezz al Arab study which revealed that, active trachoma was 1.89 times more common in rural communities compared to urban in Menofiya governorate.

In this study positive Immunoglobulin G in rural setting accounts 63.2% while in urban areas it accounts for 36.8%. However, positive Immunoglobulin M in rural areas accounts for 48.6% versus 51.4% in urban areas. This is different from Berhane et al, (2007) [7] who studied the prevalence of active trachoma by area of residency and found that the prevalence of active trachoma is more common in the rural population as compared to the urban one. This could be explained by the finding that almost half of the studied group 49.3% had recurrent infection and 50.3% of them received previous topical treatment.

In this study the prevalence of trachoma among female was higher than males 80% and 63% for Immunoglobulin M and Immunoglobulin G respectively with a significant relationship between Immunoglobulin G and female sex. This was in agreement with Courtright & West (2004)[8] who found a high prevalence of active trachoma in female due to prolonged contact of women with children in active infection during child bearing age.

Around three-quarters of the studied group were females representing 77.2% while males 22.8%, this goes in harmony with Khanduja (2012)[9] as his sample included one thousand females in underdeveloped parts of an Indian villages. Both studies showed approximately a prevalence of trachoma of 31% (in this study 31.1% while in Khanduja was 31.8%). Regarding the educational status of the studied group the majority were illiterates 65.2%. There is a statistical significance between anti-chlamydia trachomatis Immunoglobulin M and educational state (P value=0.03) where there were high level of positive Immunoglobulin M among illiterates 75.7% while non-illiterates were 24.3% and this goes in harmony with Roba et al (2013)[10] study where elderly illiterate women remain at risk of becoming blind from trachoma. Three quarter of our studied group were not working 76.8% and this could be explained by that the majority were females in comparison to Koizumia et al, (2005)[11] who found in his study that regard to mother's occupation, 48% of the studied population in his study were housewives, As for father's occupation, 75% reported having a job.

Among the 38 cases that show positive anti-chlamydial Immunoglobulin G in tears the major presentation was discharge by 42.1% followed by lacrimation in 23.7%, conjunctival congestion (red eye) in 21.1%, pain in 10.5% and itching in 2.6% and this is in harmony with Tarabishy & Jeng, (2008) [12] who stated that bacterial conjunctivitis is common and occur in patients of all ages. Typical signs are red eye and purulent discharge that persists throughout the day. Also Epling (2010) [13] stated that bacterial cause is more likely if there is gluing of the eyelids and no itching. Also the study done by Mannis & Plotnik, (2005) [14] agrees with this study as their Patients usually complain of a red eye while itching was uncommon.

Cases that showed positive anti-chlamydia Trachomatis Immunoglobulin M 27.1% of them presented with redness, 21.4% with discharge, 24.3% with lacrimation, 25.7% presented with pain and 1.4% presented with foreign body sensation and these findings confirm that Chidambaram et al, (2009)[15] who described that the condition begins slowly as inflammation of the tissue lining the eyelids (conjunctivitis, or “pink eye”).

Anti-chlamydia trachomatis Immunoglobulin G was found to be positive in 12.6%cases and negative in 87.4% and Immunoglobulin M was positive in 23.2% this goes parallel with Tabbara et al, (2000) [16] who stated that the appearance of antichlamydial antibodies in sera and tears of patients with trachoma after eye infections is well documented, specific antibodies of Immunoglobulin M, Immunoglobulin G and Immunoglobulin A classes were detected.

Immunoglobulin M of C.trachomatis in tears was found to be negative in 76.8% of patients. Mahon and Manuselis, (2000)[17] stated that Following an initial infection with C.trachomatis, the tears antibody response is usually of the Immunoglobulin M type that appears in two weeks and persists for a period of four to eight weeks. The Immunoglobulin G antibody usually appears late and persists for longer periods.

## Conclusion

Chlamydia Trachomatis is a common problem among patients especially married females and illiterates living in rural areas. Applying SAFE strategy by Family physicians in collaboration with ophthalmologists will help to control Chlamydia Trachomatis. Further studies and surveys are needed at national level in EGYPT to detect the most affected areas and the actual magnitude of the problem.
